# Determinants of policy innovation diffusion in China’s provincial sports industry funds: A mixed-methods analysis of strategic adaptation and institutional constraint

**DOI:** 10.1371/journal.pone.0354242

**Published:** 2026-08-21

**Authors:** Dongyue Wei, Zhe Ren, Hongtao Yu

**Affiliations:** 1 Yantai Nanshan University, Yantai, China; 2 School of Kinesiology and Health, Capital University of Physical Education and Sports, Beijing, China; University of Naples Federico II: Universita degli Studi di Napoli Federico II, ITALY

## Abstract

Existing policy-diffusion theories emphasize economic capacity, market maturity, and intergovernmental pressure, yet these factors may operate differently in specialized industrial policies. This study examines the diffusion of provincial sports industry development funds in China through a mixed-methods design combining discrete-time event history analysis of 31 provincial-level administrative regions from 2006 to 2022 with thematic analysis of 30 stakeholder interviews. The results show that fiscal self-sufficiency and sports lottery sales are positively associated with policy adoption, underscoring the importance of fiscal discretion and policy-specific resources. By contrast, the number of sports enterprises is negatively associated with adoption, contrary to the hypothesized positive relationship and consistent with a tentative compensatory interpretation in which dedicated funds respond to perceived industrial weakness. Administrative pressure is significant in several reduced models but not in the fully specified model, suggesting that national directives shape the institutional context without independently determining provincial adoption timing. Interview findings further reveal how strategic resource allocation, symbolic responses to public demand, formal compliance, and selective policy adaptation mediate the translation of policy conditions into local action. Together, the findings advance a context-sensitive account of diffusion organized around resource specificity, compensatory intervention, and strategic local translation, thereby identifying important boundary conditions of conventional policy-diffusion explanations.

## 1. Introduction

The institutionalization of specialized funding mechanisms has emerged as a critical lever in China’s strategic pivot toward sports industrialization, operationalizing national blueprints including the Healthy China 2030 agenda and the 14th Five-Year Plan’s sport-for-development paradigm. Unlike conventional sectoral subsidies, these development funds embody a sophisticated policy instrument that strategically couples macroeconomic objectives with micro-level behavioral incentives – catalyzing infrastructure modernization, enterprise innovation, and mass participation through market-conforming interventions. This financial engineering transcends mere fiscal allocation; it constitutes an institutional innovation reconstituting state-market-society relations within China’s transitional political economy, positioning sports as both a social good and an emergent growth vector within national development matrices [1].

Policy diffusion has been extensively examined in the literature and refers to the process through which policy adoption by one unit, such as a country, state, or city, influences other units to adopt similar policies [2]. Scholars have identified several mechanisms driving this process, including learning, competition, coercion, and emulation [3,4]. Learning occurs when policymakers observe the successes and failures of policies implemented elsewhere and adapt these insights to their own contexts [5]. Competition drives policymakers to adopt innovative policies to remain competitive. In sport, research has highlighted the role of mega-events like the Olympics in shaping national sports policies and promoting global policy learning and innovation [6–8]. Yet such frameworks inadequately explain the variegated adoption patterns observed in China’s provincial systems, where stark inter-regional resource disparities fundamentally mediate policy mobility. Grounded in resource-based view theory, our study introduces a capacity-constraint diffusion model that reconstitutes policy adoption as a function of jurisdictional resource portfolios [9].

Policy-diffusion scholarship commonly distinguishes between vertical mechanisms, through which higher-level authorities influence subnational policy choices, and horizontal mechanisms, through which jurisdictions learn from or compete with their peers [10]. In China’s multilevel governance system, however, these mechanisms are closely intertwined. Provincial governments respond to national policy priorities while retaining discretion in selecting, adapting, and implementing specific policy instruments. External signals may therefore establish a common policy agenda without producing uniform provincial responses. Differences in adoption timing and policy design may depend on how provincial actors interpret central directives in relation to local fiscal capacity, industrial conditions, and administrative priorities. This study consequently conceptualizes the diffusion of sports industry development funds as an interaction between institutional pressures and strategic local adaptation rather than as the automatic transmission of either vertical mandates or horizontal policy models.

To examine this interaction, the study constructs a province-year dataset covering 31 provincial-level administrative regions from 2006 to 2022 and applies discrete-time event history analysis to evaluate eight explanatory variables across policy-subject, policy-target, and policy-environment dimensions. Semi-structured stakeholder interviews are incorporated to explore how policymakers, implementers, and beneficiaries interpret decision-making processes, peer influence, and implementation constraints. By integrating these two forms of evidence, the study examines not only which provincial conditions are associated with policy adoption but also how institutional signals and local priorities may be translated into particular policy responses. The study thereby tests the applicability and boundary conditions of conventional policy-diffusion explanations in the context of specialized sports industry funding policies.

## 2. Literature review

The diffusion of policy innovation, especially in the context of the sports industry, is a multifaceted phenomenon that has garnered significant academic attention. Understanding how policy innovations spread and impact the sports industry, particularly with special funds aimed at development, is crucial for optimizing policy design and implementation.

### 2.1. Diffusion of innovation theory

The Diffusion of Innovation Theory, developed by Everett Rogers in 1962, is a key framework that explains how innovations—including new ideas, products, or practices—spread within a social system over time. According to this theory, the diffusion of innovations is a social process involving four main elements: innovators, communication channels, time, and social systems. Innovators are the individuals who first adopt an innovation; they typically have higher risk tolerance and socio-economic status. Communication channels refer to how information about the innovation is transmitted, which can include mass media or interpersonal communication. The time dimension refers to the entire process from the first introduction of the innovation to its widespread adoption, typically passing through five stages: knowledge, persuasion, decision, implementation, and confirmation. Finally, the structure, norms, and values of the social system significantly influence the acceptance and spread of innovations. The Diffusion of Innovation Theory has been widely applied across various fields, including public health, education, and marketing, to understand how innovations are adopted and spread [11–13].

In the sports sector, the effectiveness of policies within the sports industry, such as health promotion initiatives or programs aimed at increasing access to sports facilities, is closely tied to public acceptance and the scale of government promotional efforts. These factors collectively determine the success of policy implementation and the broader adoption of innovations within the industry [14]. In China, local governments have effectively stimulated the growth of the sports industry through innovative policies, including tax incentives and targeted funding support. However, the diffusion rate and effectiveness of these policy innovations vary considerably across different regions. These differences are influenced by factors such as the degree of government support, levels of socio-economic development, and cultural context [15].

### 2.2. Policy innovation diffusion

The exploration of how policy innovations spread began in the United States during the 1960s, drawing from fields like communication studies, sociology, and geography. In recent decades, this area has undergone significant transformation, becoming an essential domain within policy sciences as both a phenomenon to observe and a theoretical framework for analyzing policy processes. Innovation diffusion can be categorized into three distinct phases: the initial phase (1969–1990) concentrated on theories based on single factors; the second phase (1990–2000) saw the emergence of integrated theories; while the third phase (2000-present) is marked by advancements in comprehensive models [16]. The current body of literature can be distilled into three primary themes:

#### 2.2.1. Characteristics of policy innovation diffusion.

The diffusion of policy innovations demonstrates distinct temporal and spatial patterns, providing valuable insights into how policies spread and are adopted. A common feature is the S-shaped curve, which reflects a trajectory of gradual initial adoption, rapid uptake, and eventual stabilization, capturing the dynamic nature of the process. Additionally, diffusion often follows a proximity effect, where neighboring regions are more likely to adopt similar policies, and a hierarchical leader-follower effect, where early adopters influence others to follow [17]. Rogers (1995) expanded on these findings by classifying adopters into groups such as innovators, early adopters, and laggards, demonstrating that the adoption process can be represented by both S-shaped (cumulative) and bell-shaped (non-cumulative) curves. An R-shaped curve, depicting non-linear diffusion patterns observed in specific policy contexts, has also been identified in the literature [18]. Additionally, steep S-shaped curves and stepwise patterns highlight both gradual and rapid adoption scenarios, often resembling epidemic-like spreads [19]. These patterns highlight the multifaceted mechanisms underlying policy diffusion, emphasizing both regional and systemic influences on the adoption process.

#### 2.2.2. Models and mechanisms of policy innovation diffusion.

The diffusion of policy innovations, particularly within federal systems, can be explained through several theoretical models. Key frameworks include national interaction, regional dissemination, leader-follower dynamics, and vertical influence, which capture different pathways of policy diffusion [20]. Horizontal diffusion, occurring among similar entities, and vertical diffusion, involving interactions across government levels, provide further differentiation in understanding diffusion mechanisms [4]. Political ideology and party alignment have been shown to significantly influence these patterns, with partisan networks serving as critical channels for spreading innovations [21]. Moreover, local governments frequently act as catalysts for state-level policy adoption, underscoring the bottom-up dynamics that often drive diffusion processes [22].

The mechanisms that drive policy diffusion have also been extensively examined. Walker (1969) initially proposed that learning, competition, and electoral pressure are critical drivers. More recent studies highlight emulation, where jurisdictions adopt policies from others to enhance legitimacy or improve efficiency, as a critical mechanism [23,24]. These mechanisms reflect the strategic considerations of policymakers as they navigate the interplay between innovation benefits and local contextual constraints. Ideological factors, particularly polarization, further influence diffusion, acting as either a catalyst or barrier depending on the issue and political context [25].

#### 2.2.3. Factors influencing policy innovation diffusion.

Initial research on the diffusion of policy innovation primarily focused on either internal or external factors, categorized as the internal and external factor models. Internal factors, such as a government’s economic conditions, industrial capacity, level of urbanization, and financial resources, significantly influence its propensity to adopt new policies. In contrast, external factors encompass pressures from higher levels of government, intergovernmental competition, and societal influences. Mechanisms such as intergovernmental interactions, participation in conferences, and professional networks further facilitate diffusion by leveraging institutional linkages and collaborative platforms [26].

Recognizing the limitations of analyzing internal or external factors in isolation, Berry and Berry (1990) proposed an integrated approach, combining these dimensions through event history analysis to examine state lottery policy diffusion in the United States. Expanding on this, broader systems-based frameworks categorize influences into three domains: the characteristics of the innovation itself, the attributes of adopting entities, and the surrounding social and institutional context. These frameworks provide a more comprehensive and nuanced understanding of the diffusion process [27].

Recent studies have further refined these frameworks. Studies demonstrate that economic stress and political leadership interact to drive policy innovations, reflecting the dynamic interplay of state-specific conditions and external pressures. [28]. Additionally, network analysis has revealed the importance of geographic proximity and ideological alignment in shaping state policy adoption, emphasizing the intricate balance between local conditions and broader systemic influences [29].

### 2.3. Special funds for the development of the sports industry

While the term Special Funds for the Development of the Sports Industry is not widely recognized internationally, there is considerable research on how governments financially support the sports sector. The academic literature on this topic can be broadly categorized into two main areas: the use of tax policies to foster the development of the sports industry and the provision of direct financial subsidies.

#### 2.3.1. Tax policies to promote the sports industry.

Tax policies aimed at promoting the sports industry demonstrate diverse governmental strategies to foster sports infrastructure and activities. In the United States, municipal bonds are commonly employed to finance sports facility projects, with tax exemptions frequently applied to incentivize investment in these facilities [30]. Comparative analyses between the U.S. and Canada highlight the extensive use of both one-time and ongoing tax breaks to subsidize stadium construction and event operations, demonstrating a long-standing reliance on fiscal incentives in this sector [31]. Notably, more than three-quarters of sports venues built since 2000 in the U.S. benefited from federal tax-exempt bonds, though the broader economic impact of these subsidies remains contested. In the United Kingdom, studies have explored the impact of tax policy on investment, showing that changes in tax policy can affect the cost of capital and investment decisions [32]. In China, targeted tax reductions have significantly boosted private investment in sports infrastructure, with such policies also driving urbanization and regional development by integrating sports initiatives into broader economic planning [33].

#### 2.3.2. Financial subsidies to support the sports industry.

Government subsidies play a pivotal role in supporting the sports industry by addressing both operational sustainability and infrastructure development. In Australia, public financial support is directed toward wage subsidies for professional sports teams and funding for sports venues, with the level of subsidy often determined by the scale of events hosted [34]. Beyond elite sports, subsidies also target grassroots participation, reflecting a dual focus on fostering high-level performance and broad community engagement. In Sweden, a long-established system of public subsidies has been shown to provide significant support to voluntary sports associations. This funding framework reflects a well-organized collaboration between national and local governments and the Swedish Sports Confederation, enabling over 20,000 clubs to foster public health and civic engagement [35].Research from Canada underscores the broader social and economic impact of government subsidies for major sporting events. Evidence indicates that such funding significantly boosts local economies, generates tourism, and enhances community engagement, showcasing the multifaceted benefits of these investments [36]. These findings collectively highlight the importance of direct government funding as a critical mechanism for supporting the sports industry, particularly in advancing competitive sports, youth development, and broader socio-economic objectives.

## 3. Research methods

### 3.1. Data collection

The data for this study were drawn from authoritative and reliable sources to ensure analytical rigor and precision in examining the diffusion of policy innovations related to the Special Funds for the Development of China’s Sports Industry. Primary data were sourced from the China Statistical Yearbooks, which provide comprehensive statistics on economic, social, and sector-specific variables across provinces, including public expenditures, demographic trends, GDP, and investments in the sports industry. Provincial government reports were analyzed to trace the allocation and utilization of the special funds, offering insights into regional policy adoption and implementation. Socioeconomic indicators from the National Bureau of Statistics of China ensured cross-regional consistency and comparability, while government announcements and policy documents provided valuable information on the timing, structure, and progression of the policy’s implementation.

The analysis covers 31 provincial-level administrative regions in mainland China, excluding Hong Kong, Macao, and Taiwan. The observation period extends from 2006 to 2022. The year 2006 provides a pre-adoption baseline, while the first recorded provincial adoption occurred in Beijing in 2007. The province–year data were organized as a discrete-time risk set: each province contributed annual observations until the year of its first recorded adoption or until the end of the observation period if no adoption occurred. After its first adoption, a province exited the risk set and contributed no subsequent observations. To preserve temporal ordering and reduce potential simultaneity, selected time-varying covariates were entered using their one-year-lagged values.

### 3.2. Model specification

This study employs an Event History Analysis (EHA) model to investigate the factors influencing the diffusion of China’s special funds policy for sports industry development, following Berry and Berry’s (1990) pioneering application to policy innovation, such as state lottery adoption. EHA, a standard econometric tool for dynamic processes, examines transitions from non-adoption to adoption of the policy at the provincial level, as seen in prior Chinese studies on fiscal reforms and residence permit systems. The event is defined as a province’s first recorded adoption of the policy. Province–years prior to adoption remain in the risk set. Given the absence of precise adoption dates, we adopt a discrete-time hazard model with year as the observation unit, estimated via binary logistic regression in Stata 17.0. The hazard function, denoted as λ(t), represents the probability of policy adoption at time *t*. The logit-transformed model is:


$$\begin{array}{c} \begin{array}{cc} \text{log}\left(\frac{\lambda (t)}{\left(\text{1}-\lambda (t\right)}\right) & =\beta_{\text{0}}+\beta_{\text{1}}X_{\text{1}}(t-\text{1})+\beta_{\text{2}}X_{\text{2}}(t-\text{1})+\beta_{\text{3}}X_{\text{3}}(t-\text{1})+\beta_{\text{4}}X_{\text{4}}(t)+\beta_{\text{5}}X_{\text{5}}(t-\text{1})\\ & +\beta_{\text{6}}X_{\text{6}}(t)+\beta_{\text{7}}X_{\text{7}}(t)+\beta_{\text{8}}X_{\text{8}}(t)+\varepsilon (t) \end{array} \end{array}$$ (1)

To capture time-varying covariates in a province-year panel dataset, Equation 1 integrates economic and institutional drivers while addressing potential reverse causality through one-period lags for select variables (t-1). The independent variables are: $X_{\text{1}}(t-\text{1})$ Real per capita GDP (inflation-adjusted); $X_{\text{2}}(t-\text{1})$: Fiscal self-sufficiency ratio; $X_{\text{3}}(t-\text{1})$: Sports lottery sales; $X_{\text{4}}(t)$: Number of sports enterprises; $X_{\text{5}}(t-\text{1})$: Public demand for sports; $X_{\text{6}}(t)$: Competitive pressure; $X_{\text{7}}(t)$: Learning pressure; $X_{\text{8}}(t)$: Administrative pressure. The left-hand side represents the log-odds of adoption probability, while ε(t) serves as the time-varying error term to account for unobserved heterogeneity, ensuring a robust analysis of policy diffusion dynamics akin to frameworks in Walker [16] and Gray [37].

### 3.3. Research and variable measurement

#### 3.3.1. Dependent variable.

Policy adoption is measured as a binary event variable. A province is coded 1 only in the first year in which an official provincial document formally established or authorized an eligible special fund policy for sports industry development; all preceding at-risk years are coded 0. Consistent with the risk-set structure described in Section 3.1, provinces exit the sample upon their first adoption. Provinces for which no adoption was identified remain coded 0 in all at-risk years through 2022. Adoption years were determined through systematic annual searches of provincial government portals and the official websites of provincial sports and finance departments. Candidate documents were identified using terms including “sports industry guiding fund,” “special fund for sports industry development,” “guiding fund for sports industry development,” and “special fund for sports career development.” A document was classified as an adoption only when it was issued by an authorized provincial body and explicitly established or authorized an eligible provincial funding instrument. The year of the first qualifying document was recorded as the province’s adoption year.

#### 3.3.2. Independent variables.

The independent variables are chosen to reflect the key factors hypothesized to influence the adoption of the special funds policy for the development of the sports industry. These variables include economic, financial, social, and external pressures. All variables are measured, as shown in Table 1.

**Table 1:** Main Variables Measurement and Data Sources.

Variable Type	Variable Name	Variable Description	Data Source	Hypothesis
Dependent Variable	Policy Adoption	1 indicates that the provincial government adopts the special funding policy for sports industry development, 0 otherwise	Official websites of provincial governments	——
Independent Variables (Policy Subject Factors)	Real GDP per capita*	Real gross regional product per capita (inflation-adjusted)	National Bureau of Statistics of China	H1
	Fiscal Self-Sufficiency Ratio*	(Fiscal Revenue/ Fiscal Expenditure) × 100%	National Bureau of Statistics of China	H2
	Lottery Sales*	Sports lottery sales in each province	China Sports Lottery Yearbook	H3
Independent Variables (Policy Target Factors)	Number of Sports Enterprises	Number of sports enterprises in a province in a certain year	Tianyancha Database	H4
	Public Demand for Sports*	Population density of each province during the observation year, as a proxy for sports participation demand.	National Bureau of Statistics of China	H5
Independent Variables (Policy Environment Factors)	Competitive Pressure	Proportion of geographically adjacent provinces that have adopted the special funding policy for sports industry development.	Official websites of provincial governments	H6
	Learning Pressure	Ratio of provinces that have adopted the policy to the total number of provinces (31).	Official websites of provincial governments	H7
	Administrative Pressure	Number of relevant directives or policy documents issued by the State Council, General Office of the State Council, and General Administration of Sport within a year.	PKU Law Database	H8

Note: *denotes the variable lagged by one period.

Real Per Capita GDP, which is inflation-adjusted gross domestic product per person to account for price changes and provide a more accurate measure of economic output by avoiding inflation distortions, serves as a primary indicator of provincial economic capacity [38] and directly determines fiscal resources for policy innovation. Higher real per capita GDP fundamentally enables provincial adoption of sports funding policies by transforming general economic capacity into specific policy-implementation resources: elevated economic output directly expands fiscal reserves through taxation and fiscal autonomy—as demonstrated in Portugal where municipal sports financing relied strictly on local fiscal capabilities—thereby alleviating the core constraint of policy experimentation [39]. Concurrently, enhanced economic resources strengthen institutional capacity to execute complex funding mechanisms, evidenced by China’s finding that economic scale dictates sports policy diffusion through administrative mobilization efficiency [40]. Crucially, this economic-policy nexus operates via targeted resource-channeling wherein developed provinces prioritize sports investments as empirically validated in specialized initiatives such as demonstration cooperatives where policy efficacy was contingent on economic-priority alignment [41]. Building on these theoretical and empirical foundations, Per Capita GDP emerges as a critical factor influencing the adoption of innovative funding policies in the sports industry.

Hypothesis 1: Provinces with higher levels of Real Per Capita GDP are more likely to adopt special funds policies for sports industry development.

The fiscal self-sufficiency ratio, operationalized as the proportion of local government revenue relative to total expenditures, serves as a critical indicator of subnational financial autonomy. This metric captures the extent to which jurisdictions can mobilize internal resources without relying on intergovernmental transfers, thereby granting them enhanced discretion in resource allocation and policy experimentation [42]. In the context of the sports industry, by reducing dependency on centralized funding structures, fiscally autonomous jurisdictions can test novel instruments—including performance-based subsidy systems and public-private partnership models—without being constrained by homogenized budgetary guidelines from upper-level governments. For instance, local governments with robust fiscal capacities have established specialized funds to provide directed subsidies to sports enterprises undergoing service-oriented transformation, particularly in emerging sectors like sports tourism and health services [43]. Concurrently, fiscally autonomous jurisdictions leverage their discretion to implement innovation-driven policy frameworks that integrate technological advancements with cultural preservation, such as digital infrastructure upgrades for sports facilities combined with localized heritage initiatives to stimulate public participation [44]. Based on these insights, the following hypothesis is proposed:

Hypothesis 2**:** Provinces with higher fiscal self-sufficiency ratios are more inclined to adopt innovative policies for special funds supporting the development of the sports industry.

Sports lottery sales generate self-reinforcing fiscal cycles that directly enable innovative policy adoption. First, by funding tax incentive frameworks, specifically cost-plus-deduction policies for sports enterprises, that lower innovation barriers [45]. Second, they enable targeted investments in grassroots initiatives, including mass sports promotion [46], thereby ensuring direct alignment between fiscal resources and core policy objectives [47]. Third, lottery-driven revenue growth necessitates regulatory adaptation to new market dynamics—as seen when China’s blockchain-integrated ticket systems compelled real-time policy updates for fraud prevention and consumer protection. Crucially, this process transforms lottery revenue from a passive fiscal reservoir into an active lever for policy experimentation and implementation.

Hypothesis 3: Provinces with higher sports lottery sales are more likely to implement innovative policies for special funds supporting the development of the sports industry.

The number of sports enterprises drives the diffusion of sports industry special funds through demand-side pull, institutional capacity, and network effects [48]. High-density regions such as Zhejiang and Fujian foster collaborative ecosystems where enterprises collectively advocate for tailored fiscal support, including tax incentives for equipment manufacturers, while institutional feedback mechanisms like Fujian’s Sports Industry Development Council align funding allocation with sector needs. [49]. Entrepreneurial clusters including Shenzhen’s sports e-commerce ecosystem amplify policy diffusion via peer learning and benchmarking, enabling successful models like PPP stadium funding to spread regionally through inter-provincial cooperation platforms [50]. This creates a policy laboratory effect, where dense networks reduce transaction costs and accelerate the replication of specialized funding practices across jurisdictions. We therefore proposed:

Hypothesis 4: Provinces with a higher number of sports enterprises are more likely to adopt innovative policies for special funds supporting the development of the sports industry.

Public demand for sports refers to the preferences and potential needs of residents for sports participation, fitness services, sporting events, and related goods and facilities [51]. Growing public interest in health, leisure, and physical activity may place pressure on provincial governments to expand sports services and introduce financial instruments that support the development of the sports industry. Previous research suggests that stronger demand for accessible facilities and participation opportunities can encourage governments to adjust infrastructure investment and funding allocation [52–53]. Because directly comparable annual data on sports participation and household sports expenditure were not consistently available for all 31 provincial-level regions over the observation period, this study uses provincial population density as an indirect proxy for the concentration of potential public demand. Population density does not directly measure residents’ preferences or actual participation; rather, it captures the concentration of potential users and the corresponding pressure on sports facilities and services. On this basis, the following hypothesis is proposed:

Hypothesis 5: Provinces with higher public demand for sports are more likely to adopt innovative policies for special funds supporting the development of the sports industry.

Competitive pressure among provinces often manifests as yardstick competition, where local governments benchmark their performance against neighboring regions in areas like economic development and public services, including the sports industry [54]. This dynamic encourages officials to monitor and respond to peers’ achievements to avoid lagging in regional development and comparative performance assessments, and to strive for advantages in evaluations by higher authorities and career advancement. For instance, innovative funding mechanisms for sports venues in one province pressure others to adopt similar policies, like establishing special funds or tax incentives. This mirrors U.S. municipal competition for professional teams through publicly subsidized stadiums [55]. In China, rivalry to host events like the 2021 National Games drove Shaanxi Province to invest heavily in venues and infrastructure across multiple cities, promising economic growth despite debates over fiscal sustainability. Such inter-jurisdictional competition thus spurs facility investment and policy innovation to gain a competitive edge. Consequently, the study hypothesizes:

Hypothesis 6: Provinces under higher competitive pressure are more likely to adopt innovative policies for special funds supporting the development of the sports industry.

Learning pressure arises when provincial governments voluntarily observe and emulate successful policy approaches from peer regions, often to refine their own strategies in response to shared economic or social challenges [56]. Unlike administrative pressure, learning pressure operates horizontally, driven by self-initiated benchmarking and adaptation rather than hierarchical directives [57]. For instance, officials might scrutinize how a neighboring province has effectively allocated special funds to boost sports tourism, then adapt similar mechanisms to mitigate risks and improve outcomes in their own context. This process of evidence-based adaptation—monitoring proven practices in comparable areas—helps reduce uncertainty, sharpen decision-making, and promote policy efficacy [4]. We therefore proposed:

Hypothesis 7: Provinces experiencing higher levels of learning pressure are more likely to adopt special funding policies for sports industry development.

Administrative pressure, defined as the influence exerted by higher-level authorities on local governments through formal directives, is a key driver of policy adoption in China’s governance system [58]. In this study, administrative pressure is measured by the annual number of central directives and policy documents relevant to sports industry development, which serves as a proxy for the intensity of vertical policy signals. Although this system efficiently disseminates policy priorities—aligning provinces with national objectives like sports-led growth—it frequently obscures the boundary between mandating compliance and sparking innovation, as local officials might treat directives as inflexible commands rather than prompts for originality. Central orders for sports funding, for example, enforce standardization but may erode local autonomy by imposing uniform frameworks that disregard regional disparities in capabilities or demands [59]. Administrative pressure mitigates uncertainty via accountability mechanisms, permitting measured innovation exemplified by adaptive funds for quota management. It can also reduce flexibility, forcing rote conformity that overlooks context-specific needs and breeds inefficiency [4]. Navigating this interplay—central guidance versus local leeway—aims to uphold national uniformity in sports policy while accommodating tailored adjustments. Accordingly, the study hypothesizes:

Hypothesis 8: Provinces facing higher administrative pressure are more likely to adopt innovative policies for special funds supporting the development of the sports industry.

#### 3.3.3. Qualitative interviews and analysis.

To unravel the micro-mechanisms of policy diffusion, semi-structured interviews were conducted with 30 key stakeholders, including policymakers, implementers, and beneficiaries from 15 provinces (2022–2023). Participants were selected through a hybrid strategy combining purposive and snowball sampling. Initial interviewees were identified based on their direct engagement with sports industry fund policies, with criteria requiring policymakers to have ≥3 years of design experience, implementers to manage ≥2 provincial projects, and beneficiaries to represent entities receiving sustained funding. However, these strict thresholds may have limited inclusion of less experienced or atypical voices that could provide contrasting insights into diffusion processes. Referrals from initial participants expanded the sample until theoretical saturation was achieved, determined by the point when no new themes emerged during iterative analysis. All interviews were conducted in Mandarin Chinese and lasted between 45 and 90 minutes. Eighteen participants were interviewed in person, while 12 were interviewed via secure video conferencing. The interviews covered topics such as decision-making processes, peer influence, and implementation challenges.

Interview data were analyzed using thematic analysis [60], supported by NVivo 12. The analysis involved familiarization with the transcripts, initial coding, development and review of candidate themes, and refinement of the final thematic structure through member checking. Meaning-bearing segments of the interview transcripts served as the coding units, while the complete interview transcript served as the unit for comparing themes across participants. Coding was used to identify recurring themes and explanatory mechanisms rather than to estimate the prevalence of particular views among participants or provinces. Accordingly, coding frequencies were not interpreted or reported as population-level percentages. All qualitative interpretations and illustrative quotations reported in Section 4.5 were derived from these 30 interviews. The quotations were used to illustrate the identified mechanisms and were not treated as evidence of how frequently those mechanisms occurred across participants or provinces. The mixed-methods design integrated the quantitative patterns identified through event history analysis with the explanatory themes developed from the stakeholder interviews.

All 30 participants provided written informed consent before the interviews. The 18 participants interviewed in person signed paper consent forms, while the 12 participants interviewed remotely signed electronic consent forms. Before any interview recording began, the remote participants additionally reaffirmed their consent orally, and this confirmation was audio-recorded and documented. The study was conducted in accordance with the Declaration of Helsinki and approved by the Institutional Review Board of Yantai Nanshan University (GW2021−006). The approved protocol covered both interview modes and their associated consent and recording procedures, including remote interviewing, the use of electronic consent forms, oral reaffirmation of consent, and audio recording.Interview transcripts were de-identified by removing direct personal identifiers and assigning anonymous participant codes. Audio recordings and de-identified transcripts were securely stored on a password-protected university server accessible only to the research team. The reporting of the qualitative component was guided by the COREQ checklist.

## 4. Results

### 4.1. Descriptive statistics and correlation analysis

Table 2 provides a detailed summary of the descriptive statistics for all variables, including means, standard deviations, minimums, and maximums. These statistics reveal substantial heterogeneity across regions, particularly in terms of economic and policy-related factors such as real per capita GDP, fiscal self-sufficiency, and sports lottery sales. The variability observed in these indicators highlights disparities in regional economic capacity, which likely influence the ability to adopt and implement policy innovations. Similarly, the range of values for variables related to the sports industry, such as the number of sports enterprises and public sports demand, underscores differences in industry scale and population concentration and potential demand for sports services, offering important context for subsequent analyses.

**Table 2:** Descriptive Statistics of Variables.

Variable	N	Mean	Std.Dev.	Min	Max
Policy Adoption	376	0.0479	0.214	0.00	1.00
Real Per Capita GDP*	376	10.2781	0.617	8.56	12.07
Fiscal Self-sufficiency Rate*	376	0.4726	0.196	0.06	0.95
Sports Lottery Sales*	376	12.9774	1.018	8.65	15.84
Number of Sports Enterprises	376	8.0346	1.141	3.37	10.40
Public Demand for Sports*	376	0.0382	0.057	0.00	0.30
Learning Pressure	376	0.2286	0.299	0.00	1.00
Competitive Pressure	376	0.2483	0.217	0.00	0.58
Administrative Pressure	376	0.6649	1.014	0.00	4.00

Notes: Real per capita GDP, sports lottery sales, and the number of sports enterprises are natural-log transformed. Real per capita GDP, fiscal self-sufficiency, sports lottery sales, and public demand for sports are measured using one-year-lagged values. The table reports 376 at-risk province–year observations.

Table 3 presents the correlation analysis results for the adoption of sports industry development special fund policies, revealing varied relationships between policy adoption and the explanatory variables. Policy adoption is significantly and positively correlated with sports lottery sales, administrative pressure, and real per capita GDP. Fiscal self-sufficiency shows a moderate positive correlation at the 0.05 significance level, while the number of sports enterprises exhibits a weak positive correlation at the 0.10 significance level. However, policy adoption is not significantly correlated with public demand for sports or competitive pressure, providing no preliminary bivariate support for H5 and H6. These bivariate results indicate that neither the population-density proxy for public demand nor competitive pressure has a clear unadjusted association with policy adoption; their relationships are examined further in the multivariate models. The high correlation between sports lottery sales and the number of sports enterprises (r = 0.888) also warrants further assessment of multicollinearity, as reported in Section 4.2. Overall, the mixed correlation patterns provide a foundation for the subsequent multivariate analysis, particularly for distinguishing institutional and financial determinants from market-oriented factors in the diffusion of sports industry fund policies. The results also highlight the need for caution when applying general policy diffusion theories to specialized policy contexts such as sports industry funding programs.

**Table 3:** Correlation Matrix of Variables.

Variable	Policy Adoption	Real Per Capita GDP	Fiscal Self-sufficiency Rate	Sports Lottery Sales	Number of Sports Enterprises	Public Demand for Sports	Competition Pressure	Learning Pressure	Administrative Pressure
1.Policy Adoption	1								
Real Per Capita GDP	0.113^**^	1							
Fiscal Self-sufficiency Rate	0.107^**^	0.399^***^	1						
Sports Lottery Sales	0.151^***^	0.662^***^	0.396^***^	1					
Number of Sports Enterprises	0.086^*^	0.628^***^	0.448^***^	0.888^***^	1				
Public Demand for Sports	0.017	0.480^***^	0.691^***^	0.212^***^	0.241^***^	1			
Competitive Pressure	0.034	0.581^***^	−0.005	0.325^***^	0.218^***^	0.313^***^	1		
Learning Pressure	0.092^*^	0.719^***^	−0.192^***^	0.547^***^	0.490^***^	0.015	0.656^***^	1	
Administrative Pressure	0.136^***^	0.196^***^	−0.048	0.208^***^	0.116^**^	0.002	0.157^***^	0.281^***^	1

Notes: *means P < 0.1, **means P < 0.05, ***means P < 0.01. Correlations are Pearson coefficients. Blank cells above the diagonal are symmetric to below.

### 4.2. Multicollinearity tests

To ensure the accuracy of regression results, independent variables must remain relatively independent. Multicollinearity diagnostics are essential, commonly using the Variance Inflation Factor (VIF). Per Gujarati and Porter (2009), VIF > 10 indicates severe issues that could distort estimates. In this study, all VIF values are below 10 (mean VIF = 4.45), suggesting no severe multicollinearity, as detailed in Table 4.

**Table 4:** Results of Multicollinearity Tests.

Variable	VIF	1/VIF
Learning Pressure	7.30	0.136
Real Per Capita GDP*	5.85	0.170
Number of Sports Enterprises*	5.77	0.173
Sports Lottery Sales*	5.71	0.175
Fiscal Self-Sufficiency	4.75	0.210
Public Demand for Sports	2.73	0.366
Competitive Pressure	2.37	0.422
Administrative Pressure	1.13	0.885
Mean VIF	4.45	/

Note: *indicates that the variable is natural-log transformed. VIF values below 10 indicate that severe multicollinearity is not present, although values above 5 suggest moderate collinearity.

Despite acceptable VIFs, the correlation matrix reveals notable pairwise correlations. Higher VIFs indicate moderate multicollinearity, potentially inflating standard errors. As a supplementary exploratory diagnostic, principal component analysis (PCA) was used to examine the underlying correlation structure among the explanatory variables, with results in Table 5. PCA extracted two main components: the first captures socio-economic development and pressure factors (high loadings on Learning Pressure, Per Capita GDP, Competitive Pressure, Sports Lottery Sales, and Number of Sports Enterprises), while the second reflects fiscal and market demand factors (high loadings on Fiscal Self-Sufficiency and Public Demand for Sports). These components explain most variance without the original inter-correlations, as shown in Table 6.

**Table 5:** PCA Results – Eigenvalues, Variance Explained.

Component	Eigenvalue	Variance Explained (%)	Cumulative Variance Explained (%)
1	3.63	45.6	45.6
2	1.66	20.9	66.3
3	1.08	13.6	79.9
4	0.95	11.9	91.8
5	0.29	3.6	95.4
6	0.19	2.4	97.8
7	0.10	1.3	99.1
8	0.07	0.9	100.0

Note: KMO = 0.661, Bartlett’s test of sphericity significant (p < 0.001).

**Table 6:** Rotated Component Loadings for the First Two Principal Components.

Variable	Component 1	Component 2
Number of Sports Enterprises	0.660	0.492
Sports Lottery Sales	0.741	0.417
Fiscal Self-Sufficiency	0.010	0.953
Public Demand for Sports	0.136	0.792
Real Per Capita GDP	0.813	0.414
Learning Pressure	0.930	0.193
Competitive Pressure	0.717	0.016
Administrative Pressure	0.412	0.180

### 4.3. Regression analysis

Hypotheses were tested in Stata 17.0 using binary logistic regression within a discrete-time event history framework. Each province contributed annual observations while it remained at risk of adoption and exited the risk set after its first recorded adoption. The regression results are reported in Table 7. Models 1–3 separately examine policy-subject, policy-target, and policy-environment factors. Model 4 combines policy-subject and policy-target factors, Model 5 combines policy-subject and policy-environment factors, and Model 6 combines policy-target and policy-environment factors. Model 7 is the fully specified model incorporating all eight explanatory variables across the three groups of factors. Hypothesis evaluation is based primarily on Model 7, while Models 1–6 show how the estimated relationships change across different combinations of explanatory variables. Table 7 reports model-based standard errors, whereas Section 4.4 examines the sensitivity of the results to province-clustered standard errors and other alternative specifications.

**Table 7:** Results of the Model Regression Analysis.

Variable	Model1	Model 2	Model 3	Model 4	Model 5	Model 6	Model 7
Real Per Capita GDP	−0.0172 (0.5949)			0.9466 (0.7999)	−0.8071 (1.1225)		−0.3348 (1.2406)
Fiscal Self-sufficiency Rate	1.2399 (1.3384)			3.5473^**^ (1.7146)	4.2400 (2.7102)		7.3619^**^ (3.1214)
Sports Lottery Sales	0.8456^**^ (0.3950)			1.6126^**^ (0.6467)	0.5871 (0.4326)		1.4840^**^ (0.7025)
Number of Sports Enterprises		0.4497^*^ (0.2689)		−1.2077^**^ (0.5284)		0.3258 (0.3384)	−1.4305^**^ (0.5940)
Public Demand for Sports		−0.3533 (4.0411)		−14.3249^*^ (8.0470)		0.9236 (4.6158)	−13.1657 (8.6067)
Competitive Pressure			−0.4679 (0.9599)		−1.1728 (1.0681)	−0.4259 (1.1156)	−1.1382 (1.2232)
Learning Pressure			1.7218 (1.4488)		3.6503 (3.0854)	0.9037 (1.8146)	5.1068 (3.3532)
Administrative Pressure			0.3585^*^ (0.1877)		0.3351^*^ (0.1955)	0.3847^**^ (0.1902)	0.2859 (0.2018)
_cons	−14.7238^***^ (5.3728)	−6.6903^***^ (2.2604)	−3.6974^***^ (0.4538)	−25.6427^***^ (7.5121)	−5.6914 (11.1452)	−6.2270^**^ (2.6210)	−12.2358 (12.0799)
*N*	376	376	376	376	376	376	376
*Pseudo R* ^2^	0.0786	0.0225	0.0470	0.1316	0.1181	0.0564	0.1721

Notes: Entries are binary logit coefficients, with model-based standard errors reported in parentheses. N denotes the number of at-risk province–year observations. *** p < 0.01, ** p < 0.05, * p < 0.10.

The policy-subject factors exhibit differentiated relationships with policy adoption. In Model 7, fiscal self-sufficiency is positively and significantly associated with adoption (β = 7.3619, SE = 3.1214, p < 0.05), supporting H2. Sports lottery sales are also positively and significantly associated with adoption (β = 1.4840, SE = 0.7025, p < 0.05), supporting H3. These findings indicate that provincial fiscal discretion and policy-specific financial resources are important enabling conditions for the establishment of sports industry development funds. By contrast, real per capita GDP is not statistically significant in the fully specified model (β = −0.3348, SE = 1.2406), and H1 is therefore not supported. This result suggests that general economic development does not independently explain differences in provincial adoption once more directly deployable fiscal resources and the remaining explanatory factors are considered.

The policy-target factors reveal a more complex pattern. Although the number of sports enterprises is positively associated with adoption in the reduced Model 2, its coefficient becomes negative and statistically significant in Model 7 (β = −1.4305, SE = 0.5940, p < 0.05). Because H4 predicts a positive relationship, this result does not support H4. The negative coefficient nevertheless raises the possibility of a compensatory-adoption pattern in which provinces with a comparatively weaker enterprise base have stronger incentives to establish dedicated funds to stimulate industrial development. This interpretation is post hoc and should be treated cautiously because the proposed compensatory mechanism is not directly tested by the regression model. Public demand for sports is negative but statistically non-significant in Model 7 (β = −13.1657, SE = 8.6067); H5 is therefore not supported in the fully specified model.

The policy-environment factors likewise provide limited evidence of independent relationships in the fully specified model. Competitive pressure is negative and statistically non-significant (β = −1.1382, SE = 1.2232), while learning pressure is positive but statistically non-significant (β = 5.1068, SE = 3.3532). H6 and H7 are therefore not supported. Administrative pressure is positively and significantly associated with adoption in several reduced specifications, but the coefficient becomes statistically non-significant after all explanatory variables are included in Model 7 (β = 0.2859, SE = 0.2018). H8 consequently does not receive support in the fully specified model. The change across specifications suggests that administrative pressure may operate as a contextual or conditional influence rather than as an independent determinant of provincial adoption timing. Overall, the results indicate that policy-specific fiscal resources are more consistently associated with adoption than general economic capacity, market conditions, or intergovernmental pressures.

### 4.4. Sensitivity analyses

To assess whether the findings from the fully specified model were sensitive to alternative methodological choices, three additional analyses were conducted within the discrete-time event history framework. First, Model 7 was re-estimated using standard errors clustered at the provincial level to account for within-province dependence across annual observations. Second, a centered linear duration term was added to Model 7 to account for temporal variation in the baseline hazard of policy adoption. Third, real per capita GDP, fiscal self-sufficiency, sports lottery sales, and public demand for sports were lagged by two years rather than one year. All sensitivity specifications retained the explanatory variables included in Model 7.

The province-clustered and linear-duration specifications used the same 376 province-year observations as Model 7. Because constructing the two-year-lagged variables required excluding the first available observation for each province, the effective sample size of the two-year-lag specification was reduced to 345 province-year observations. All three specifications retained the same 18 policy-adoption events. As shown in Table 8, the coefficient for the number of sports enterprises remained negative across all three specifications. It was statistically significant in the province-clustered specification and marginally significant in both the linear-duration and two-year-lag specifications. These results indicate that the negative direction of the relationship is relatively stable, but they do not support the positive relationship predicted by H4. Administrative pressure remained positively signed but statistically non-significant in all three specifications. Therefore, H8 was not supported by the sensitivity analyses.

**Table 8:** Sensitivity Analyses Compared with Model 7.

Variable	Province-clustered Model 7	Linear duration term	Selected covariates lagged t − 2
Real Per Capita GDP	−0.3348 (1.263)	1.232 (2.164)	−0.450 (1.132)
Fiscal Self-Sufficiency	7.3619** (3.182)	4.360 (4.254)	6.769** (2.915)
Sports Lottery Sales	1.4840* (0.816)	1.456** (0.740)	1.082 (0.769)
Number of Sports Enterprises	−1.4305** (0.680)	−1.199* (0.717)	−1.152* (0.654)
Public Demand for Sports	−13.1657* (7.160)	−14.671** (7.408)	−10.853* (6.370)
Competitive Pressure	−1.1382 (1.113)	−0.869 (1.204)	−0.827 (1.132)
Learning Pressure	5.1068 (3.753)	11.363* (6.016)	4.291 (3.609)
Administrative Pressure	0.2859 (0.210)	0.172 (0.236)	0.181 (0.228)
Pseudo-R²	0.172	0.187	0.125
N	376	376	345

Notes: Province-clustered standard errors are reported in parentheses for all specifications. The linear-duration specification includes a centered duration term. In the two-year-lag specification, real per capita GDP, fiscal self-sufficiency, sports lottery sales, and public demand for sports are lagged by two years. The smaller sample in the two-year-lag specification results from excluding the first available observation for each province. *** p < 0.01, ** p < 0.05, * p < 0.10.

Fiscal self-sufficiency remained positive and statistically significant in the province-clustered and two-year-lag specifications but became statistically non-significant after the linear duration term was introduced. Sports lottery sales also remained positive, although its statistical significance varied across specifications. Overall, the sensitivity analyses provide stronger evidence regarding the direction of several relationships than regarding the stability of their statistical significance.

### 4.5. Micro-mechanisms of policy diffusion: Qualitative evidence from stakeholder interviews

The qualitative follow-up focuses primarily on four predictors that were statistically non-significant in the fully specified model: real per capita GDP (H1), public demand for sports (H5), competitive pressure (H6), and learning pressure (H7). Learning pressure nevertheless exhibited a weak positive bivariate correlation at the 0.10 significance level, but this relationship was not sustained after the remaining covariates were included. The results for H4 and H8 require a different analytical interpretation. H4 produced a statistically significant relationship in the direction opposite to that hypothesized, whereas H8 was positive in several reduced models but statistically non-significant in the fully specified model and the sensitivity analyses. Accordingly, the purpose of this section is not to provide qualitative confirmation for every unsupported hypothesis, but to examine possible mechanisms for which the interview materials provided directly relevant evidence.

This section draws on the thematic analysis of semi-structured interviews with 30 stakeholders, including policymakers, implementers, and beneficiaries. Consistent with the procedures described in Section 3.3.3, meaning-bearing transcript segments served as the coding units, while complete interview transcripts served as the units for comparing themes across participants. The qualitative evidence is used to illustrate possible mechanisms rather than to estimate the prevalence of particular views among participants or provinces. The principal themes and illustrative evidence are summarized in Table 9. All quotations were translated from Mandarin Chinese into English by the authors and checked against the original transcripts.

**Table 9:** Thematic Coding of Strategic Local Governance Behaviors.

Theme	Sub-theme	Illustrative Quote	Linked Hypothesis	Mechanistic Explanation
Resource Diversion	Trophy Project Prioritization	“Economic growth provides resources, but we prioritize projects that deliver quick political returns...” (Interviewee #7, 2023)	H1 was not supported in the full model.	Resource misallocation disconnects policy from public needs
Performative Response	Demand-Signal Manipulation	“When public demand is high, we build large complexes to demonstrate action, but actual fitness sites are neglected.” (Interviewee #12, 2023)	H5 was not supported in the full model.	High visibility projects inflate metrics (e.g., participation rates) while obscuring resource mismatches; transforms demand into symbolic compliance.
Label Compliance	Budget Reclassification	“Competition leads us to relabel old expenditures to meet superior assessments.” (Interviewee #18, 2023)	H6 was not supported in the full model or sensitivity analyses.	Competitive pressure may generate formal evidence of responsiveness without producing substantively new policy commitments.
Selective Mimicry	Model Repurposing	“We retained the ‘digital payment’ branding but redirected funds to align with leadership priorities.” (Interviewee #5, 2023)	H7 was not supported in the fully specified model.	Adopts “safe” peer models (e.g., Jiangsu’s digital currency) but strips equity mechanisms to serve commercial/political agendas.
Public-Private Bundling	Goal Hybridization	“E-sports arenas are rebranded as ‘health-tech hubs’... this secures funding under dual policy agendas.” (Interviewee #21, 2023)	H7 was not supported in the fully specified model.	Conflates commercial interests (e-sports subsidies) with public health goals via semantic reframing.

Notes: Themes and subthemes were developed through thematic coding of the 30 interview transcripts in NVivo 12. The table presents illustrative quotations and does not report the prevalence of themes among participants or provinces. Quotations are identified using anonymized participant codes and interview years. All quoted material derives from interviews conducted under the IRB-approved protocol (GW2021−006).

Although the theoretical framework predicted a positive relationship between economic development and policy adoption, real per capita GDP was not statistically significant across the regression specifications. The interview accounts provide one possible explanation: greater economic capacity does not necessarily translate into a stronger commitment to sports industry fund policies. Participants described how some provincial governments prioritized highly visible and commercially oriented projects, such as e-sports arenas and sports tourism complexes, because these projects were perceived to generate more immediate political or economic returns. Such prioritization may divert attention and resources from less visible community fitness programs, thereby weakening the expected relationship between economic development and policy adoption. As one interviewee explained, “Economic growth provides resources, but we prioritize projects that deliver quick political returns rather than addressing grassroots needs” (Interviewee #7, 2023). This account illustrates a possible mechanism of strategic resource allocation and policy decoupling; it does not establish the prevalence of this behavior across provinces.

Public demand for sports was hypothesized to be positively associated with policy adoption. However, its coefficient in the full model was negative but statistically non-significant, while the sensitivity specifications produced consistently negative estimates that were statistically significant or marginally significant. Because these estimates were opposite to the positive relationship predicted by H5, they did not support the hypothesis. The interview accounts suggest that expressions of public demand may sometimes be translated into highly visible projects, such as sports tourism developments, rather than less visible community-based fitness services. Such responses may not correspond to the forms of sports provision that residents demand in their daily lives, potentially weakening the expected relationship between public demand and policy adoption. As one interviewee explained, “When public demand is high, we build large complexes to demonstrate action, but actual fitness sites are neglected” (Interviewee #12, 2023). This account illustrates how a visible policy response may symbolically acknowledge public demand without necessarily strengthening the substantive connection between demand and policy adoption. It therefore offers a possible interpretation of the statistically non-significant and model-dependent result rather than evidence of a systematic negative effect.

Interprovincial competition was also expected to promote policy adoption. However, competitive pressure was not statistically significant in either the full model or the sensitivity analyses reported in Section 4.4. The interview accounts suggest that symbolic compliance may provide one possible explanation. Some participants described instances in which existing expenditures were relabeled or reclassified to demonstrate compliance with higher-level assessments without necessarily creating substantively new policy commitments. As one interviewee explained, “Competition leads us to relabel old expenditures to meet superior assessments” (Interviewee #18, 2023). Such relabeling may create formal evidence of policy responsiveness without a corresponding increase in substantive policy innovation or resource commitment. These accounts suggest that when administrative evaluations emphasize visible responses, competitive pressure may encourage formal compliance rather than substantive innovation. This mechanism offers one possible interpretation of why competitive pressure was not significantly associated with policy adoption in the quantitative models.

Learning pressure showed a weak positive bivariate correlation at the 0.10 significance level. However, the coefficient was no longer statistically significant after administrative pressure and the other covariates were included in the full model. The interview accounts suggest that selective policy adaptation may provide one possible explanation for this model-dependent result. Some participants described how recognizable elements of policies adopted elsewhere were retained while their implementation was adjusted to local political and economic priorities. As one interviewee explained, “We retained the ‘digital payment’ branding but redirected funds to align with leadership priorities” (Interviewee #5, 2023). A related interview account described how commercially oriented projects could be reframed to align with both industrial-development and public-health objectives (Interviewee #21, 2023). These accounts suggest that policy learning may involve selective reinterpretation rather than direct replication, with greater attention given to policy elements that can be aligned with visible industrial or administrative priorities. This mechanism may help explain why the weak bivariate association between learning pressure and policy adoption was not sustained in the full model. The interview evidence is interpreted as illustrating a possible mechanism of selective adaptation rather than establishing how frequently this behavior occurred across provinces.

Taken together, the qualitative findings suggest that the lack of support for the hypothesized positive relationships should not be interpreted as evidence that the underlying policy mechanisms are irrelevant. Instead, economic resources, public demand, interprovincial competition, and policy learning may be strategically reinterpreted during provincial implementation. Resource allocation may favor highly visible projects, expressions of public demand may be translated into symbolic policy responses, competitive pressure may encourage formal compliance, and policy learning may involve selective adaptation rather than direct replication.

The H4 and H8 results further reinforce this interpretation of conditional and strategically mediated diffusion. The negative coefficient for the number of sports enterprises is inconsistent with the positive relationship predicted by H4 but may indicate a compensatory pattern in which provinces with a comparatively weak enterprise base have stronger incentives to introduce dedicated policy support. Because the interviews were not designed to estimate the prevalence of this mechanism, this interpretation remains tentative. Similarly, the attenuation of the administrative-pressure coefficient in the fully specified model suggests that national directives may provide a common institutional background without independently determining differences in provincial adoption timing. These findings identify boundary conditions of conventional policy-diffusion explanations rather than invalidating the broader theoretical framework.

## 5. Research conclusions and countermeasures

### 5.1. Research conclusions and discussion

Conventional policy-diffusion explanations generally assume that economically stronger jurisdictions, more mature target markets, and greater intergovernmental pressure are more likely to adopt policy innovations. The findings of this study qualify that expectation. In the diffusion of provincial sports industry development funds, adoption was not uniformly associated with greater economic development, a larger sports-enterprise base, stronger public demand, or greater horizontal and vertical pressure. Instead, the results reveal three more differentiated dynamics: the importance of deployable policy-specific resources, the possibility of compensatory policy intervention, and the strategic local translation of institutional signals.

First, the results distinguish general economic capacity from resources that can be directly mobilized for a specialized policy instrument. Real per capita GDP did not demonstrate an independent relationship with adoption, whereas fiscal self-sufficiency and sports lottery sales were positively and significantly associated with adoption in the fully specified model, supporting H2 and H3. Both variables retained positive coefficients across the sensitivity specifications. These findings suggest that aggregate wealth and deployable policy capacity should not be treated as equivalent: a province may possess substantial economic resources without allocating them to a sports industry fund, whereas fiscal discretion and sports-related revenue can be mobilized more directly. This interpretation connects policy-diffusion research on socioeconomic capacity [61] with fiscal federalism research emphasizing subnational discretion. The interview accounts provide a complementary explanation for the non-significant GDP result by indicating that general economic resources may sometimes be redirected toward projects offering greater political visibility or more immediate commercial returns rather than converted into dedicated funding commitments.

Second, the policy-target results challenge the assumption that a larger enterprise base necessarily precedes government intervention. The number of sports enterprises was negative and statistically significant in Model 7 and remained negative across the sensitivity specifications. H4 was therefore not supported in its hypothesized positive direction. One possible post hoc interpretation is a compensatory-adoption logic: provinces with a comparatively weak enterprise base may have stronger incentives to establish dedicated funds to stimulate industrial development, whereas provinces with more mature markets may rely more heavily on existing commercial and financial networks. This interpretation is relevant to research on the dynamic transformation of sports industry services, but it remains tentative because the analysis did not directly test the proposed compensatory mechanism and the enterprise measure overlaps empirically with other indicators of provincial resource capacity. Public demand for sports likewise did not demonstrate the positive independent relationship predicted by H5. Interview participants described how expressions of demand could be translated into highly visible construction or promotional projects rather than community-based services, suggesting that demand is filtered through local definitions of political visibility and administrative feasibility. This interpretation is consistent with research showing that the effects of government sports expenditure depend on how resources are configured and allocated.

Third, the policy-environment findings suggest that institutional pressures operate as contextual and strategically mediated influences rather than automatic adoption triggers. Competitive and learning pressures did not exhibit independent relationships in the fully specified model. Administrative pressure was positive and significant in several reduced models but became statistically non-significant after all explanatory factors were included in Model 7 and remained non-significant in the sensitivity analyses. H8 therefore did not receive support as an independent determinant of adoption. Nevertheless, national directives may still place sports industry development on provincial agendas and define legitimate policy instruments without independently explaining why some provinces adopt earlier than others. The qualitative findings illuminate this distinction. Accounts of expenditure relabeling suggest that competition may encourage formal compliance without producing substantively new commitments, while accounts of selective adaptation indicate that provinces may retain recognizable policy language but redirect implementation toward local political and commercial priorities.

Complementing the regression analysis, the thematic analysis of 30 stakeholder interviews identifies strategic resource allocation, symbolic responses to public demand, formal compliance, and selective policy adaptation as possible mechanisms connecting institutional conditions to provincial responses. These themes are not used as independent tests of the hypotheses or as estimates of how frequently particular practices occurred among participants or provinces. Rather, they provide an actor-centered account of how economic resources, social demand, competition, and policy learning may be interpreted and reframed during local implementation. The qualitative and quantitative components therefore perform complementary functions: the regression analysis evaluates the hypothesized relationships, while the interview evidence helps illuminate the strategic processes through which policy conditions are translated into provincial responses.

Taken together, the findings suggest a resource–compensation–translation interpretation of policy diffusion in this specialized field. Adoption depends more clearly on resources that provincial governments can discretionarily mobilize than on general economic development; industrial policy may respond to perceived market weakness as well as market maturity; and external policy signals are filtered through strategic local adaptation rather than transmitted automatically into substantive adoption. The contribution of this study therefore lies not in maximizing the number of statistically supported hypotheses, but in identifying the boundary conditions of a uniform, additive model of policy diffusion. By showing how deployable fiscal resources, perceived industrial needs, and local interpretation interact, the study offers a more context-sensitive explanation of policy innovation diffusion within China’s decentralized yet hierarchically coordinated governance system.

### 5.2. Policy recommendations

To address the identified challenges in policy diffusion, this study proposes targeted and feasible measures that build on existing governance structures in China’s sports sector.

First, to counter fiscal incentives that skew resources toward commercial projects, the central government could establish a national sports industry development fund administered by the General Administration of Sport. This fund would prioritize resource allocation to underdeveloped regions, such as those in the western and northeastern provinces, through needs-based grants allocated according to regional fiscal capacity and community access needs. Such an initiative is politically practical, as it can be piloted through central-local partnerships to ease fiscal pressures on lagging areas while promoting greater local autonomy and reducing long-term dependency on direct subsidies, thereby fostering balanced regional development as supported by recent studies on economic disparities.

Second, to mitigate selective mimicry and policy homogenization, local authorities should introduce digital monitoring systems to track policy adaptation and implementation, using standardized indicators that compare stated policy commitments with documented fund allocations, actual disbursements, beneficiaries, and implementation outcomes. Complementing this, inter-regional talent exchanges and training programs could encourage context-sensitive innovations, with rollout leveraging established e-governance platforms for cost efficiency and incentives rewarding effective implementations to ensure broad stakeholder engagement.

Finally, to bridge enforcement gaps and resolve vertical-horizontal conflicts, intergovernmental coordination could be strengthened via collaborative forums backed by clear legal delineations of responsibilities and integrated information-sharing networks, drawing on international best practices for enhanced accountability. Anchoring these in national policy directives would facilitate rapid adoption, minimizing redundancies and supporting adaptable, sustainable diffusion aligned with broader goals of equitable sports governance.

## 6. Research limitations and future prospects

While this study offers valuable insights into the mechanisms and challenges of policy innovation diffusion in the sports industry, several limitations warrant attention. First, the findings are based on province-level observational data from China, which may limit their generalizability to countries or governance systems with different administrative and fiscal structures. Second, the study primarily focuses on structural and institutional aspects of intergovernmental coordination, with limited consideration of informal networks and cultural dynamics that often influence policy diffusion. Third, the econometric models rely on total values for certain variables (e.g., sports lottery sales and number of sports enterprises) rather than per capita real values, which may introduce scale bias in cross-provincial comparisons, as larger provinces naturally exhibit higher totals that could exaggerate their influence. To address these limitations, future research could explore municipal-level variation, expand the qualitative sample, and conduct comparative case studies to further examine these mechanisms, such as how trust, organizational culture, and informal collaborations shape policy outcomes. Additionally, adopting per capita or otherwise scale-adjusted measures for key variables could serve as an interesting direction for future studies to test the robustness of our conclusions, potentially revealing whether scale-normalized measures alter the direction or significance of relationships. Future work should also broaden the geographical and institutional scope to test these findings in diverse contexts, including developing economies, and extend the observation period and examine post-adoption implementation trajectories to evaluate the longer-term effects of policy interventions. By addressing these limitations, future work can provide a more comprehensive framework for understanding policy diffusion, contributing to more effective and sustainable governance strategies in the sports industry and beyond.

## Supporting information

S1 File Data. (XLSX)
